# Cell Death and Metabolic Stress in *Gymnodinium catenatum* Induced by Allelopathy

**DOI:** 10.3390/toxins13070506

**Published:** 2021-07-20

**Authors:** Leyberth José Fernández-Herrera, Christine Johanna Band-Schmidt, Tania Zenteno-Savín, Ignacio Leyva-Valencia, Claudia Judith Hernández-Guerrero, Mauricio Muñoz-Ochoa

**Affiliations:** 1 Centro Interdisciplinario de Ciencias Marinas (IPN-CICIMAR), Av. Instituto Politécnico Nacional s/n, Col. Playa Palo de Santa Rita, La Paz 23096, Mexico; cguerrer@ipn.mx (C.J.H.-G.); mmunozo@ipn.mx (M.M.-O.); 2 Centro de Investigaciones Biológicas del Noroeste (CIBNOR), S.C. Instituto Politécnico Nacional 195, Col. Playa Palo Santa Rita, La Paz 23096, Mexico; tzenteno04@cibnor.mx; 3 Consejo Nacional de Ciencia y Tecnología-Instituto Politécnico Nacional, Centro Interdisciplinario de Ciencias Marinas (CONACyT, IPN-CICIMAR), Col. Playa Palo de Santa Rita, La Paz 23096, Mexico; ileyvava@conacyt.mx

**Keywords:** allelopathy, apoptosis, caspase-3, harmful algae, reactive oxygen species

## Abstract

Allelopathy between phytoplankton species can promote cellular stress and programmed cell death (PCD). The raphidophyte *Chattonella marina* var. *marina*, and the dinoflagellates *Margalefidinium polykrikoides* and *Gymnodinium impudicum* have allelopathic effects on *Gymnodinium catenatum*; however, the physiological mechanisms are unknown. We evaluated whether the allelopathic effect promotes cellular stress and activates PCD in *G. catenatum*. Cultures of *G. catenatum* were exposed to cell-free media of *C. marina* var. *marina*, *M. polykrikoides* and *G. impudicum*. The mortality, superoxide radical (O_2_^●−^) production, thiobarbituric acid reactive substances (TBARS) levels, superoxide dismutase (SOD) activity, protein content, and caspase-3 activity were quantified. Mortality (between 57 and 79%) was registered in *G. catenatum* after exposure to cell-free media of the three species. The maximal O_2_^●−^ production occurred with *C. marina* var*. marina* cell-free media. The highest TBARS levels and SOD activity in *G. catenatum* were recorded with cell-free media from *G. impudicum*. The highest protein content was recorded with cell-free media from *M. polykrikoides*. All cell-free media caused an increase in the activity of caspase-3. These results indicate that the allelopathic effect in *G. catenatum* promotes cell stress and caspase-3 activation, as a signal for the induction of programmed cell death.

## 1. Introduction

The succession among phytoplankton species during harmful algal bloom (HAB) events is complex, and the mechanisms of bloom-species selection and how some species dominate over others is not clear [1,2,3,4]. In allelopathic interactions, specific chemical compounds (allelochemicals) produced by one species can induce damage or benefit another species [5]. Some chemical signals between co-existing phytoplankton groups induce programmed cell death (PCD) as a selective strategy in intraspecies competition [6,7,8].

*Gymnodinium catenatum* is a marine dinoflagellate that produces paralytic shellfish toxins (PST) and forms HABs [9,10], particularly in tropical and subtropical coastal zones [11,12]. During HAB events, the co-occurrence of *G. catenatum* with the raphidophyte *Chattonella marina* var. *marina* and dinoflagellates such as *Margalefidinium polykrikoides* and *Gymnodinium impudicum,* have been reported in different geographic regions [13,14,15,16,17,18]. 

*Chattonella marina* var. *marina* and *M. polykrikoides* are known to produce reactive oxygen species (ROS) such as superoxide radical (O_2_^●−^), hydrogen peroxide (H_2_O_2_) and hydroxyl radicals (HO^●^) [19,20,21,22,23,24], as well as hemolysins, hemagglutinins and polyunsaturated fatty acids [25,26,27]. *Gymnodinium impudicum* does not produce toxins [28]; however, it excretes exopolysaccharides that can cause fish death, due to the blocking of gills [29,30,31]. The dominance between co-occurring phytoplankton species is associated in part with their nutrient uptake efficiency, light and space [32,33,34]; some species use allelopathy, which refers to the production of chemical substances that limit the growth or kill their competitors, as a competition strategy [35,36,37]. In laboratory conditions, cell-free media from *C. marina* var. *marina*, *M. polykrikoides* and *G. impudicum* have an allelopathic effect on *G. catenatum* that causes loss of flagella, cell deformation and lysis [18,38]. Although the growth inhibition in *G. catenatum* is clear, the metabolic response to the allelopathic effect is unknown. Understanding the mechanisms that promote allelopathy allows us to understand how this phenomenon is reflected in the dominance of the species [39]. 

Allelopathy can induce changes in the activation of programmed cell death (PCD) between phytoplankton species [40,41]. There are different modes of cell death in phytoplankton cells. Necrosis is a severe PCD that rapidly causes a total loss of cell integrity, and apoptosis is a process resulting from the activation of chord subsystems for self-destruction causing chromatin condensation, nucleus depletion by DNA cuts and loss of cell membrane integrity [42,43]. Apoptosis is mediated by a family of proteases with cysteine-specific protease residues of C-terminal aspartic acid, named caspases. These enzymes are divided into two groups, initiators (1, 2, 4, 5, 8, 9, 10, 11) and executors (3, 6, 7, 14) [44]. In addition, a group of proteases called metacaspases, which are similar in structure to caspases but related to a different substrate, are not directly related to PCD; in protists, the metacaspases are previously activated as death-signaling proteins [45]. Apoptosis mediated by caspases has been described in eukaryotic and prokaryotic groups, including phytoplankton such as cyanobacteria [46], diatoms [47], chlorophytes [48], dinoflagellates [49] and coccolithophores [50,51]. 

Few studies on the processes that activate PCD in bloom-forming species have been published [52]. Via allelochemicals, the cyanobacterium *Microcystis* sp. causes the collapse of *Peridinium gutanense* blooms [53]. The algicidal bacterium *Kordia algicida* secretes proteases that promote cell death in the diatoms *Skeletonema costatum*, *Thalassiosira weissflogii* and *Phaeodactylum tricornutum* [54]. Allelochemical signaling usually acts selectively on a particular phytoplankton group; *Shawanella* sp. has an algicidal effect on dinoflagellates, but not on chlorophytes or cryptophytes [55]. The main response induced by allelopathic stress in phytoplankton is the increase in ROS production, particularly H_2_O_2_ and O_2_^●−^ [53,56,57]. In addition, significant increases in the activity of antioxidant enzymes such as catalase (CAT) and superoxide dismutase (SOD), which are reflected in mitochondrial membrane potential changes, related to cytochrome C functionality, triggering PCD by the action of caspases-3 and -9, have been reported [8]. It has been suggested that allelopathy among cooperating species may be the main detonator of the ROS-PCD complex [58,59,60]. Therefore, in this study the allelopathic effect of three HAB species, *C.* *marina* var. *marina*, *G. impudicum* and *M. polykrikoides* in *G. catenatum*, was analyzed and the enzymatic activity of aspartate substrate-caspase-3, O_2_^●−^ production, SOD activity, lipid peroxidation and protein content were quantified in order to relate with PCD caused by allelopathy. 

## 2. Results

### 2.1. Growth Rates and Growth Curve Stages 

**Figure 1:**
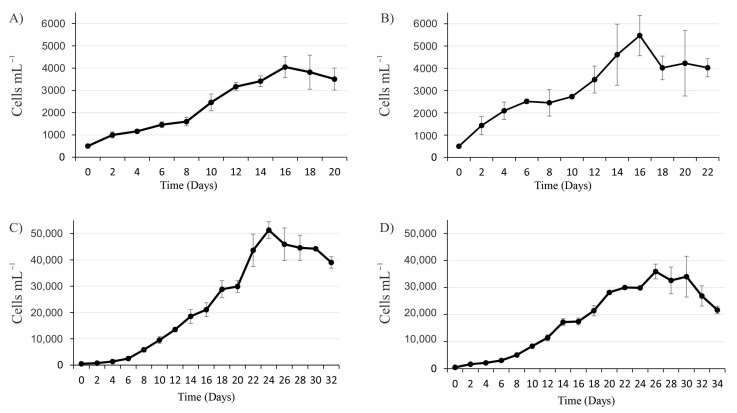
Growth curves in GSe media, (**A**) *Gymnodinium catenatum*, (**B**) *Margalefidinium polykrikoides*, (**C**) *Chattonella marina* var. *marina* and (**D**) *Gymnodinium impudicum*. Data are presented as mean ± SD, (*n* = 3).

The exponential growth rates of *G. catenatum* and *M. polykrikoides* were 0.57 div day^−1^ and 0.59 div day^−1^, respectively; the growth rate of *C. marina* var. *marina* was 0.43 div day^−1^, while for *G. impudicum* it was 0.48 div day^−1^ (Table 1). The maximum cell density obtained varied among species (Figure 1). Maximum cell density in *G. catenatum* was 4048 ± 440 cells mL^−1^, reached at the 16th day of culture. The early exponential phases (EEP) ended on the 4th day, the late exponential phase (LEP) ended on the 16th day, and the death phase (DP) initiated on the 18^th^ day of culture. The dinoflagellate *M. polykrikoides* registered a maximum density of 5470 ± 904 cells mL^−1^ at the 16th day of culture. The EEP lasted until the 4th day and the LEP lasted until the 16th day, followed by the DP. The raphidophyte *C. marina* var. *marina* was the species that reached the highest cell density of 51,324 ± 3201 cells mL^−1^ at day 24. The adaptation phase ended on day 6, on day 22 it reached the LEP, and the death phase began after the 24th day. The dinoflagellate *G. impudicum* reached a maximum cell density of 35,924 ± 2734 cells mL^−1^ at 26 days of culture, the EEP initiated on day 6, the LEP lasted from day 8 to day 24, and the DP occurred after day 28. The strains of *C. marina* var. *marina* and *G. impudicum* showed an adaptation phase between the 2nd and the 4th day of culture, a phase that was not observed in *G. catenatum* and *M. polykrikoides* strains.

**Figure 2:**
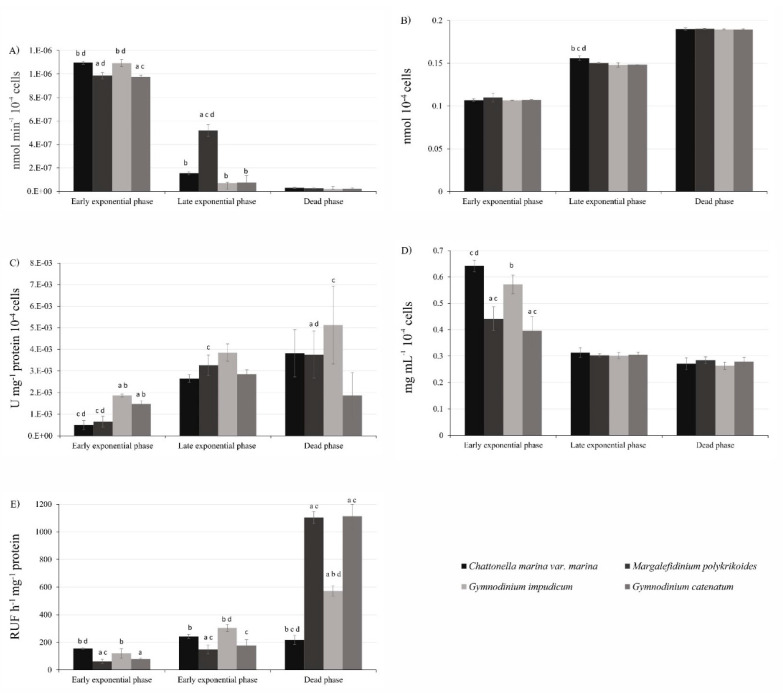
(**A**) Superoxide radical production (O_2_^●^^−^), (**B**) thiobarbituric acid reactive substances (TBARS), (**C**) superoxide dismutase (SOD) activity, (**D**) total protein concentrations, and **(E)** caspase-3 activity. Data are presented as mean ± SD, (*n* = 3). Letters represent significant differences among species (*p* < 0.05).

During the growth phases, differences in the production of metabolites and the enzyme activity were found (Figure 2). The maximum O_2_^●−^ production in all species was observed during the EEP (Figure 2A). The highest O_2_^●–^ production was found in *C. marina* var. *marina* (1.1 × 10^−6^ ± 1.26 × 10^−8^ nmol min^−1^ 10^−4^ cells), which was similar to *G. impudicum* (1.09 × 10^−6^ ± 3.32 × 10^−8^ nmol min^−1^ 10^−4^cells), followed by *M. polykrikoides* (9.9 × 10^−7^ ± 2.81 × 10^−8^ nmol min^−1^ 10^−4^ cells), and *G. catenatum* (9.7 × 10^−7^ ± 1.45 × 10^−8^ nmol min^−1^ 10^−4^ cells), with statistical differences between all species (one-way ANOVA F_3,8_ = 1.24, *p* < 0.05). In the LEP, *M. polykrikoides* produced the highest O_2_^●−^ production 5.19 × 10^−7^ ± 5.12 × 10^−8^ nmol min^−1^ 10^−4^ cells, which was three times higher than *C. marina* var. *marina* and six times higher than *G. impudicum* and *G. catenatum*, with significant differences between *M. polykrikoides* and the other species (ANOVA F_3,8_ = 5.96, *p* < 0.05). Minimal production of O_2_^●−^ was found in the DP in all species (~1.90 to 3 × 10^−8^ ± 9.46 × 10^−9^ nmol min^−1^ 10^−4^ cells), without significant differences among species (ANOVA F_3,8_ = 3.53, *p* < 0.05).

Lipid peroxidation quantified as TBARS levels increased with the culture age (Figure 2B). In EEP, *M. polykrikoides* had the highest TBARS levels (1.09 × 10^−1^ ± 5.02 × 10^−3^ nmol 10^−4^ cells), higher than *C*. *marina* var. *marina* with 1.07 × 10^−1^ ± 1.49 × 10^−3^ nmol 10^−4^ cells, followed by *G. impudicum* and *G. catenatum* with ~1.09 × 10^−1^ ± 2.50 × 10^−4^ nmol 10^−4^ cells; however, no significant differences were observed among species (ANOVA F_3,8_ = 11.35, *p* < 0.05). The raphidophyte *C. marina* var. *marina* had the highest TBARS levels during the LEP (1.56 × 10^−1^ ± 2.77 × 10^−3^ nmol 10^−4^ cells), followed by *M. polykrikoides* (1.50 × 10^−1^ ± 6.50 × 10^−4^ nmol 10^−4^ cells), *G. catenatum* (1.49 × 10^−1^ ± 2.00 × 10^−4^ nmol 10^−4^ cells) and *G. impudicum* (1.48 × 10^−1^ ± 2.56 × 10^−4^ nmol 10^−4^ cells), with a significant difference between all species (ANOVA F_3,8_ = 6.15, *p* < 0.05). In all species, the maximum production of TBARS was found in the DP; similar levels between *C. marina* var. *marina* (1.90 × 10^−1^ ± 1.54 × 10^−3^ nmol 10^−4^ cells) and *M. polykrikoides* (1.90 × 10^−1^ ± 0.50 × 10^−3^nmol 10^−4^ cells), and among *G. impudicum* (1.89 × 10^−1^ ± 0.56 × 10^−3^ nmol 10^−4^ cells), and *G. catenatum* (1.89 × 10^−1^ ± 0.83 × 10^−3^ nmol 10^−4^ cells) were found. In the DP, no significant differences were found among species (ANOVA F_3,8_ = 3.58, *p* < 0.05).

Superoxide dismutase activity increased with culture age in *C. marina* var. *marina*, *M. polykrikoides*, *G. impudicum* and *G. catenatum* (Figure 2C). The highest SOD activity was found in *G. impudicum* in the EEP (1.85 × 10^−3^ ± 7.14 × 10^−5^ U mg^−1^ protein 10^−4^ cells), followed by *G. catenatum* (1.46 × 10^−3^ ± 1.56 × 10^−4^ U mg^−1^ protein 10^−4^ cells), *C. marina* var. *marina* (4.85 × 10^−4^ ± 2.13 × 10^−4^ U mg^−1^ protein 10^−4^ cells) and *M. polykrikoides* (6.45 × 10^−4^ ± 2.52 × 10^−4^ U mg^−1^ protein 10^−4^ cells), with significant differences among all species (ANOVA F_3,8_ = 1.82, *p* < 0.05). During the LEP, the SOD highest activity was found in *G. impudicum* and *M. polykrikoides,* with 3.84 and 3.26 × 10^−3^ ± 4.44 × 10^−4^ U mg^−1^ protein 10^−4^ cells, respectively, followed by *G. catenatum* with 2.84 × 10^−3^ ± 1.99 × 10^−4^ U mg^−1^ protein 10^−4^ cells. The lowest SOD activity was found in *C. marina* var. *marina* (2.64 × 10^−3^ ± 1.76 × 10^−4^ U mg^−1^ protein 10^−4^ cells). Significant differences in the SOD activity during LEP were found in all species (ANOVA F_3,8_ = 1.85, *p* < 0.05). In the DP, *G. impudicum* showed the highest SOD activity (5.12 × 10^−3^ ± 1.81 × 10^−3^ U mg^−1^ protein 10^−4^ cell), followed by *M. polykrikoides* (3.75 × 10^−3^ ± 1.08 × 10^−3^ U mg^−1^ protein 10^−4^ cell) and *C. marina* var. *marina* with 3.81 × 10^−3^ ± 1.10 × 10^−3^ U mg^−1^ protein 10^−4^ cell. The dinoflagellate *G. catenatum* displayed the lowest SOD activity in the DP (1.85 × 10^−3^ ± 1.07 × 10^−3^ U mg^−1^ protein 10^−4^ cells). Significant differences were only recorded in *G. impudicum* with the rest of the strains (ANOVA F_3,8_ = 0.35, *p <* 0.05).

In the EEP, the maximum total protein content was recorded in *C. marina* var. *marina* (0.64 ± 0.02 mg mL^−1^ proteins 10^−4^ cells (Figure 2D), followed by *G. impudicum* (0.57 ± 0.03 mg mL^−1^ proteins 10^−4^ cells), *M. polykrikoides* (0.44 ± 0.04 mg mL^−1^ proteins 10^−4^ cells) and *G. catenatum* (0.39 ± 0.05 mg mL^−1^ proteins 10^−4^ cells), with significant differences among all species (ANOVA F_3,8_ = 0.99, *p* < 0.05). In the LEP, all species presented average values of ~0.30 ± 0.01 mg mL^−1^ proteins 10^−4^ cells (LEP, ANOVA F_3,8_ = 0.84, *p* < 0.05), and the protein concentration decreased slightly in the DP to ~0.27 ± 0.02 mg mL^−1^ proteins 10^−4^ cells, with no significant differences among them (DP, ANOVA F_3,8_ = 0.82, *p* < 0.05). 

The caspase-3 activity showed a progressive increase with the growth phases (Figure 2E). All strains showed a lower caspase-3 activity during the EEP. In this stage, the highest activity was observed in *C. marina* var. *marina* (155 ± 6 RFU h^−1^ mg^−1^ protein) and *G. impudicum* (120 ± 34 RFU h^−1^ mg^−1^ protein), while in *G. catenatum* and *M. polykrikoides* it was lower (79 ± 6 RFU h^−1^ mg^−1^ protein and 61 ± 15 RFU h^−1^ mg^−1^ protein, respectively). During the LEP, the highest caspase-3 activity was recorded in *G. impudicum* (303 ± 25 RFU h^−1^ mg^−1^ protein) and *C. marina* var. *marina* (242 ± 16 RFU h^−1^ mg^−1^ protein), followed by *G. catenatum* (178 ± 42 RFU h^−1^ mg^−1^ protein) and *M. polykrikoides* (147 ± 32 RFU h^−1^ mg^−1^ protein) (ANOVA F_3,8_ = 17.25, i < 0.05). Maximum caspase-3 activity occurred in the DP; the highest caspase activity was recorded in *G. catenatum* (1,113 ± 86 RFU h^−1^ mg^−1^ protein) and *M. polykrikoides* (1,104 ± 44 RFU h^−1^ mg^−1^ protein), followed by *G. impudicum* (572 ± 336 RFU h^−1^ mg^−1^ protein) and *C. marina* var. *marina* (218 ± 34 RFU h^−1^ mg^−1^ protein). Caspase-3 activity in all growth phases showed significant differences among all species (ANOVA F_3,8_ = 377.25, *p* < 0.05).

### 2.2. Allelopathy Experiments

**Figure 3:**
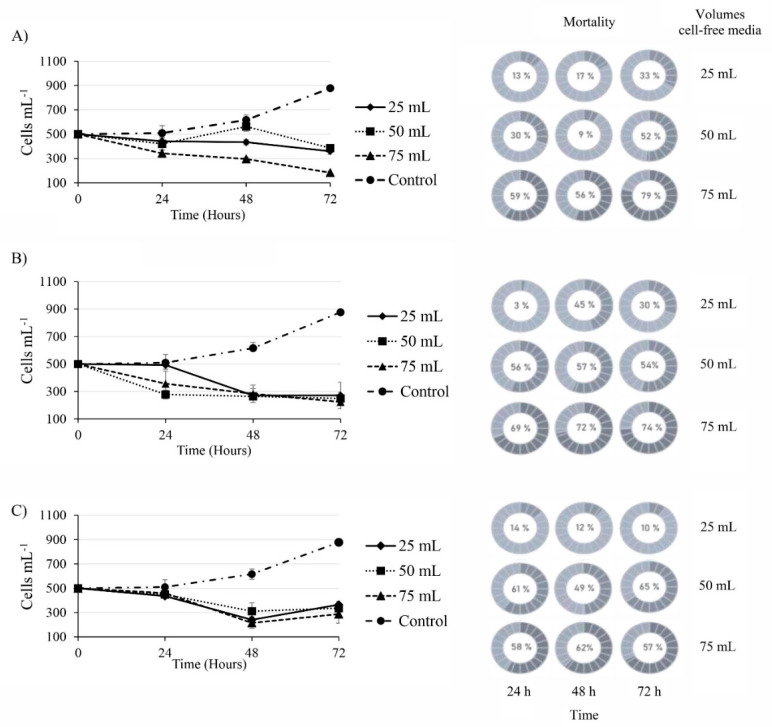
Cellular abundance of *Gymnodinium catenatum* after exposure to cell-free media from (**A**) *Chattonella marina* var. *marina*, (**B**) *Margalefidinium polykrikoides* and (**C**) *Gymnodinium impudicum*. Data are shown as mean ± SD, (*n* = 3). Circular graphics represent the mortality percentage of treatments compared to the control.

Cell-free media from the three species caused mortality in *G. catenatum* (Figure 3). The highest mortality was found when *G. catenatum* was exposed to the largest volumes (50 and 75 mL) of cell-free media (Figure 3A). When 75 mL from *C*. *marina* var. *marina* cell-free media was added, there was 79% of mortality in *G. catenatum* after 72 h. With the same volume (75 mL of cell-free medium) of *M. polykrikoides* in the same period (72 h), 74% of death in *G. catenatum* cells was observed, while *G. impudicum* caused 57% mortality at 72 h (Figure 3B,C). Conversely, 50 mL of cell-free medium from *G. impudicum* caused 65 % of mortality in *G. catenatum* cells at 72 h, while when cells were exposed to 75 mL of cell-free media, the mortality in *G. catenatum* cells was lower (62 and 57 % at 48 and 72 h, respectively) compared to the mortality caused with a volume of 50 mL (49 and 65%). The cell abundance of *G. catenatum* cultures exposed to cell-free media from *C. marina* var. *marina*, *M. polykrikoides* and *G. impudicum* decreased in less than 72 h (Figure 3A–C). Cell-free media (75 mL) from *C. marina* var. *marina* caused a maximum decrease from 500 to 184 ± 12 cells mL^−1^ in *G. catenatum* after 72 h of exposure; with the same volume of cell-free media from *M. polykrikoides*, a decrease from 500 to 224 ± 21 cells mL^−1^ occurred. When 75 mL of cell-free media of *G. impudicum* was added, the highest decrease in *G. catenatum* cells occurred at 48 h from 500 to 214 ± 44 cells mL^−1^; however, a slight increase to 287 ± 75 cells mL^−1^ was observed at 72 h, suggesting a recovering process in the cell growth. In the control treatment with their own cell-free media, cell abundance of *G. catenatum* increased from 500 to 887 ± 8 cells mL^−1^ from 0 to 72 h. In the control treatment with GSe media, the cell abundance of *G. catenatum* was similar to that reported in the growth phase 500 to 899 ± 12 cells mL^−1^ from time 0 to 72 h (data not shown by the similarity of the results).

### 2.3. Superoxide Radical (O_2_^●−^) Production 

**Figure 4:**
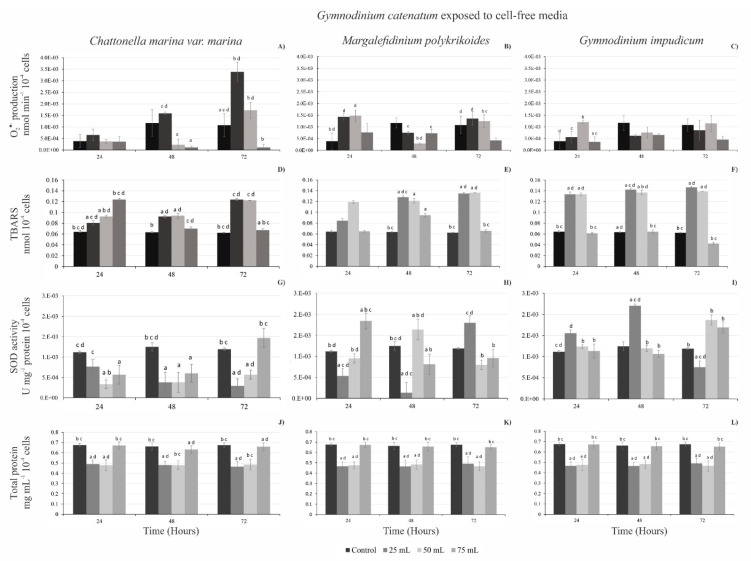
Allelopathic effects in *Gymnodinium catenatum* exposed to cell-free media from *Chattonella marina* var. *marina*, *Margalefidinium polykrikoides* and *Gymnodinium impudicum* evaluated by (**A**–**C**) superoxide radical production (O_2_^●^^−^), (**D**–**F**) thiobarbituric acid reactive substances (TBARS), (**G**–**I**) superoxide dismutase (SOD) activity, and (**J**–**L**) total protein content. Data are shown as mean ± SD. Letters represent significant differences among treatments (different volumes of cell-free media) with respect to the respective control (*p* < 0.05, *n* = 3).

The O_2_^●−^ production of *G. catenatum* exposed to exudates of cell-free media varied with species and treatments (Figure 4A–C). The maximal O_2_^●−^ production occurred when 25 mL of *C. marina* var*. marina* cell-free media was added; at 24 h there was an increase in O_2_^●−^ production, which continued until 72 h (3.38 × 10^−3^ ± 4.45 × 10^−4^ nmol min^−1^ 10^−4^ cells), followed by the addition of 50 mL (1.72 × 10^−3^ ± 3.42 × 10^−4^ nmol min^−1^ 10^−4^ cells), and when *G. catenatum* was exposed to 75 mL of cell-free media of *C. marina* var*. marina* O_2_^●−^ production was 1.14 × 10^−3^ ± 5.02 × 10^−4^ nmol min^−1^ 10^−4^ cells, being significantly lower than the control (ANOVA F_3,8_ = 11.35, *p* < 0.05). With *M. polykrikoides* 25 mL of cell-free media at 24 h, the highest O_2_^●−^ production in *G. catenatum* was 1.43 × 10^−3^ 1.40 ± × 10^−4^ nmol min^−1^ 10^−4^ cells; with 50 mL, the production was similar (1.47 × 10^−3^ ± 2.48 × 10^−4^ nmol min^−1^ 10^−4^ cells), and when adding 75 mL of cell-free media of *M. polykrikoides* it only reached a production of 0.76 × 10^−3^ ± 3.85 × 10^−4^ nmol min^−1^ 10^−4^ cells (ANOVA F_3,8_ = 11.35, *p* < 0.05). With 50 mL cell-free media of *G. impudicum* at 24 h, O_2_^●−^ production of *G. catenatum* was 1.20 × 10^−3^ nmol min^−1^ 10^−4^ cells, which was higher than when 25 mL of cell-free media was added 1.47 × 10^−3^ ± 2.48 × 10^−4^ nmol min^−1^ 10^−4^ cells, and with the addition of 75 mL it increased to 3.53 × 10^−3^ ± 1.23 × 10^−4^ nmol min^−1^ 10^−4^ cells (ANOVA F_3,8_ = 2.29, *p* < 0.05). At 48 h, the O_2_^●−^ production in all volumes added was significantly lower than the control. At 72 h, the highest O_2_^●−^ production in *G. catenatum*, was found when adding 50 mL of *G. impudicum* cell-free media 1.14 × 10^−3^ ± 5.85 × 10^−4^ nmol min^−1^ 10^−4^ cells, which was higher than when 25 mL (0.85 × 10^−3^ ± 1.59 × 10^−4^ nmol min^−1^ 10^−4^ cells) or 75 mL (0.45 × 10^−3^ ± 0.80 × 10^−4^ nmol min^−1^ 10^4^) was added. Only at 24 h were significant differences observed among treatments compared with the control (ANOVA F_3,8_ = 4.87, *p* < 0.05).

### 2.4. Thiobarbituric Acid Reactive Substances (TBARs) Levels

Lipid peroxidation in *G. catenatum* was higher when exposed to 25 and 50 mL volumes at 48 and 72 h of cell-free media from all species (Figure 4D–F). When *G. catenatum* was exposed to cell-free media from the raphidophyte at 24 h (Figure 4D) with 75 mL, the TBARS levels were higher (0.12 ± 1.73^−3^ nmol 10^−4^ cells) compared to the control (0.06 ± 0.03^−4^ nmol 10^−4^ cells); with the addition of 50 mL the TBARS levels were 0.09 ± 2.53^−3^ nmol 10^−4^ cells; and with 25 mL they decreased to 0.08 ± 4.25^−3^ nmol 10^−4^ cells (ANOVA F_3,8_ = 0.87, *p* < 0.05). The TBARS levels in *G. catenatum*, after exposure to cell-free media from the other dinoflagellate species, were similar, particularly in treatments of 25 and 50 mL, at 48 and 72 h, respectively. Cell-free media from *M. polykrikoides* caused the highest lipid peroxidation in *G. catenatum* (from 0.12 to 0.13 ± 2.45^−3^ nmol 10^−4^ cells) at 48 and 78 h with statistical differences with the control (ANOVA F_3,8_ = 4.54, *p* < 0.05). Similarly, treatments with 25 and 50 mL of cell-free media of *G. impudicum* yielded the highest TBARS levels in *G. catenatum* at 24, 48 and 72 h, with values from 0.13 to 0.14 ± 1.11^−3^ nmol 10^−4^ cells with a significant difference with the control, and when adding 75 mL of the cell-free filtrate from 0.04 to 0.6 ± 0.13^−3^ nmol 10^−4^ cells (*p* < 0.05). 

### 2.5. Superoxide Dismutase (SOD) Activity 

The SOD activity of *G. catenatum* was variable and did not describe a dose–time relationship (Figure 4G–I). The highest SOD activity in *G. catenatum* exposed to 25 mL of cell-free culture from *C. marina* var. *marina* was at 24 h with 0.76 × 10^−3^ ± 1.74 × 10^−4^ U mg^−1^ protein 10^−4^ cells with respect to 75 mL (0.50 × 10^−3^ ± 2.30 × 10^−4^ U mg^−1^ protein 10^−4^ cells) and 50 mL (0.33 × 10^−3^ ± 1.08 × 10^−4^ U mg^−1^ protein 10^−4^ cells). At 48 h, with 75 mL of cell-free culture from *C. marina* var. *marina*, the highest SOD activity was found (0.59 × 10^−3^ ± 2.19 × 10^−4^ U mg^−1^ protein 10^−4^ cells), while at 25 and 50 mL the SOD activity was similar (~ 0.37 × 10^−3^ ± 2.45 × 10^−4^ U mg^−1^ protein 10^−4^ cells) (Figure 4G). After 72 h of exposure, SOD activity in *G. catenatum* cells with 75 mL was higher (1.46 × 10^−3^ ± 2.34 × 10^−4^ U mg^−1^ protein 10^−4^ cells) compared to when adding 50 mL (0.56 × 10^−3^ ± 1.08 × 10^−4^ U mg^−1^ protein 10^−4^ cells) and 25 mL of cell-free media. The SOD activity in *G. catenatum* decreased (0.29 × 10^−3^ ± 1.68 × 10^−4^ U mg^−1^ protein 10^−4^ cells) compared to the control (one-way ANOVA, F_3,4_ = 1.78, (*p* < 0.05)). The SOD activity in *G. catenatum* when exposed to allelochemicals of *M. polykrikoides* (Figure 4H) was highest at 24 h with 75 mL of cell-free filtrate (1.84 × 10^−3^ ± 2.08 × 10^−4^ U mg^−1^ protein 10 ^−4^ cells), compared to 50 mL (0.95 × 10^−3^ ± 1.88 × 10^−4^ U mg^−1^ protein 10^−4^ cells) and 25 mL (0.53 × 10^−3^ ± 0.72 × 10^−4^ U mg^−1^ protein 10^−4^ cells). At 48 h, when adding 50 mL of the filtrate from *M. polykrikoides*, higher SOD activity (1.63 × 10^−3^ ± 1.21 × 10^−4^ U mg^−1^ protein 10^−4^ cells) was observed compared to when 75 mL was added (1.81 × 10^−3^ ± 2.39 × 10^−4^ U mg^−1^ protein 10^−4^ cells) and with 25 mL (0.13 × 10^−3^ ± 1.55 × 10^−4^ U mg^−1^ protein 10^−4^ cells). With the lowest volume (25 mL) of cell-free media from *M. polykrikoides*, at 72 h there was a higher SOD activity (1.80 × 10^−3^ ± 5.54 × 10^−4^ U mg^−1^ protein 10^−4^ cells), compared to 75 mL (0.95 × 10^−3^ ± 2.06 × 10^−4^ U mg^−1^ protein 10^−4^cells) and 25 mL (0.78 × 10^−3^ ± 1.67 × 10^−4^ U mg^−1^ protein 10^−4^cells) of cell-free media; all volumes were statistically different from the control (ANOVA, F_3, 8_ = 5.54, *p* < 0.05). In the treatments with *G. impudicum* cell-free media, the highest SOD activity was recorded at 24 h with 25 mL (1.55 × 10^−3^ ± 0.76 × 10^−4^ U mg^−1^ protein 10^−4^cells), higher than the SOD activity caused when adding 50 mL (1.24 × 10^−3^ ± 0.42 × 10^−4^ U mg^−1^ protein 10^−4^ cells) and 75 mL (1.13 × 10^−3^ ± 1.65 × 10^−4^ U mg^−1^ protein 10^−4^ cells). At 72 h, volumes of 50 and 75 mL of cell-free media from *G. impudicum* caused an SOD activity in *G. catenatum* of 1.87 × 10^−3^ ± 0.42 × 10^−4^ and 1.69 × 10^−3^ × 10^−3^ ± 0.29 × 10^−4^ U mg^−1^ protein 10^−4^ cells, respectively, higher than when adding 25 mL of the filtrate that caused an SOD activity in *G. catenatum* of 1.13 × 10^−3^ ± 1.65 × 10^−4^ U mg^−1^ protein 10^−4^ cells; in all volumes, SOD activity was statistically different from the control (ANOVA, F_3,8_ = 0.62, (*p* < 0.05)). 

### 2.6. Protein Content

Total protein content in *G. catenatum* was significantly different among the different treatments when adding cell-free media from the three species (Figure 4J–L). Treatments with 75 mL and the control had the highest protein values. At 24, 48 and 72 h when adding 75 mL of *C. marina* var*. marina* cell-free media, the highest protein concentration in *G. catenatum* (~0.65 ± 0.03 mg mL^−1^ proteins 10^−4^ cells), as compared to 25 and 50 mL of filtrate (~0.47 ± 0.04 mg mL^−1^ proteins 10^−4^ cells), was observed; the control and the treatment with 75 mL of cell-free media was statistically different from the 25 and 50 mL cell-free media treatments (ANOVA, F_3,8_ = 1.16, *p* < 0.05). With cell-free filtrates of *M. polykrikoides*, at 24, 48 and 72 h the highest protein content in *G. catenatum* was found when adding 75 mL and the control (~0.66 ± 0.02 mg mL^−1^ proteins 10^−4^ cells); when adding 25 and 50 mL of the filtrate, the protein content decreased (~0.47 ± 0.04 mg mL^−1^ proteins 10^−4^ cells). Statistical differences between the 75 mL of cell-free media with respect to the 25 and 50 mL cell-free media treatments were found (ANOVA, F_3,8_ = 1.53, *p* < 0.05). With 75 mL of *G. impudicum* cell-free medium, the protein content in *G. catenatum* was ~ 0.67 ± 0.05 mg mL^−1^ proteins 10^−4^ cells, higher than when adding 25 and 50 mL (~0.47 ± 0.05 mg mL^−1^ proteins 10^−4^ cells); statistical differences between the control and 75 mL of cell-free media compared to 25 and 50 mL of cell-free media treatments were found (ANOVA, F_3,8_ = 3.53, (*p* < 0.05)).

### 2.7. Caspase-3 Activity 

**Figure 5:**
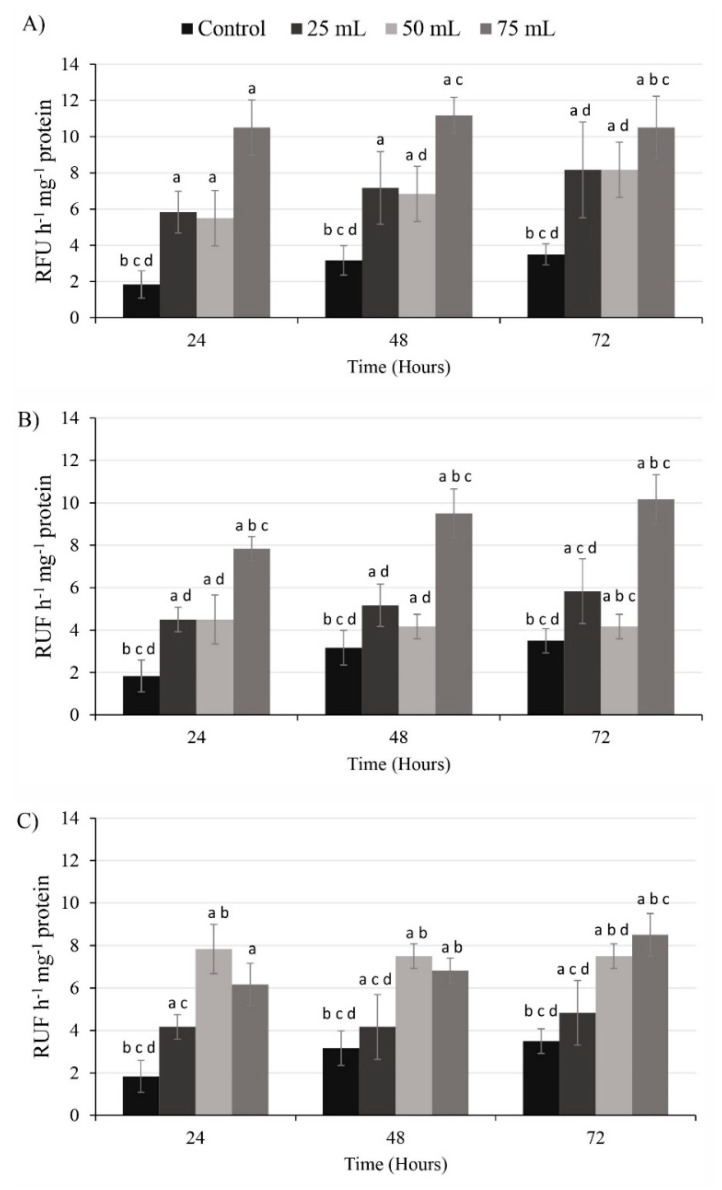
Caspase-3 activity in *Gymnodinium catenatum* exposed to cell-free media from (**A**) *Chattonella marina* var. *marina*, (**B**) *Margalefidinium polykrikoides* and (**C**) *Gymnodinium impudicum*. Data are shown as mean ± SD. Letters represent significant differences among treatments (different volumes of cell-free media) with respect to the respective control (*p* < 0.05, *n* = 3).

All cell-free media volumes of *C. marina* var. *marina*, *M. polykrikoides* and *G. impudicum* tested increased caspase-3 activity in *G. catenatum* (Figure 5). With 75 mL of cell-free media from *C. marina* var. *marina*, the highest caspase-3 activity was found at 24 h (10.5 ± 1.5 RFU h^−1^ mg^−1^ protein), compared to adding 25 and 50 mL cell-free media from *C. marina* var. *marina*, with an activity of 6 ± 1.1 and 5.5 ± 0.5 RFU h^−1^ mg^−1^ protein, respectively. After 48 h, similar values were recorded; 75 mL from *C. marina* var. *marina* cell-free media in cells of *G. catenatum* recorded maximum caspase-3 activity (11.2 ± 1 RFU h^−1^ mg^−1^ protein), higher than when adding 25 mL (7.1 ± 2 RFU h^−1^ mg^−1^ protein) and 50 mL (6.8 ± 0.05 RFU h^−1^ mg^−1^ protein). With 75 mL from *C. marina* var. *marina* cell-free media, at 72 h the caspase-3 in cells of *G. catenatum* was higher 10.5 ± 1.7 RFU h^−1^ mg^−1^ protein, compared to volumes of 25 and 50 mL with average values of 8.16 ± 1.7 RFU h^−1^ mg^−1^ protein; all volumes showed differences significant compared to control (ANOVA F_3,8_ = 1.77, *p* < 0.05). As for response to the exposure of *M. polykrikoides* cell-free filtrates, when adding 75 mL at 24 h, higher caspase-3 activity was observed in *G. catenatum* (7.8 ± 1.7 RFU h^−1^ mg^−1^ protein), compared to the activity found with 25 and 50 mL that registered ~4.56 ± 0.8 RFU h^−1^ mg^−1^ protein. After 48 h, filtrates from *M. polykrikoides* caused an increase in the caspase-3 activity in *G. catenatum* to 9.5 ± 1.5 RFU h^−1^ mg^−1^ protein with 75 mL, while with 25 and 50 mL of cell-free media the caspase-3 activity was of 5.2 ± 1 RFU h^−1^ mg^−1^ protein and 4.16 ± 0.6 RFU h^−1^ mg^−1^ protein, respectively. With 75 mL of cell-free media from *M. polykrikoides*, the highest caspase-3 activity in *G. catenatum* was recorded at 72 h (9.5 ± 1.5 RFU h^−1^ mg^−1^ protein), compared with the caspase-3 activity at 24 and 48 h with the same volume 75 mL. At 72 h, volumes of 25 and 50 mL of cell-free media of *M. polykrikoides* showed values in caspase-3 activity similar to those at 24 and 48 h (~4.9 ± 1 RFU h^−1^ mg^−1^ protein); all treatments were significantly different from the control (ANOVA F_3,8_ = 2.28, *p* < 0.05). The activity of the caspase-3 with cell-free media of *G. impudicum* varied with time; during 24 and 48 h, when adding 50 mL of media the caspase-3 activity was higher (~ 7.65 ± 0.7 RFU h^−1^ mg^−1^ protein) than when adding 25 and 75 mL, (6.2 ± 1 RFU h^−1^ mg^−1^ protein and 4.2 ± 1 RFU h^−1^ mg^−1^ protein), respectively. Significant differences were found in all treatments with the control (ANOVA F_3,8_ = 6.38, *p* < 0.05). After 72 h, the caspase activity in *G. catenatum* was higher when 75 mL cell-free media of *G. impudicum* was added (8.5 ± 1 RFU h^−1^ mg^−1^ protein), followed by 50 mL and 25 mL treatments (7.5 ± and 4.8 ± 0.5 RFU h^−1^ mg^−1^ protein), respectively, with significant differences among treatments with the control (ANOVA F_3,8_ = 1.66, *p* < 0.05). 

Correlation analyses of caspase-3 activity with molecules related to oxidative stress and the total protein content in *G. catenatum* exposed to cell-free filtrates of *C. marina* var. *marina*, *M. polykrikoides* and *G. impudicum* are shown in Table 2. The cell-free media from *C. marina* var. *marina* showed strong negative correlations between caspase-3 and O_2_^●^^−^ production in *G. catenatum* at 48 and 72 h (r = −0.796, r = −0.707, *p* < 0.05), respectively; TBARS levels had a negative correlation (r = −0.927) at 72 h (*p* < 0.05); the SOD activity during 24 and 48 h had a negative correlation (r = −0.852 and r = −0.733, respectively) (*p* < 0.05); the protein content also presented a negative correlation at 24 and 48 h (r = −0.731 and r = −0.739, respectively) (*p* < 0.05). With *M. polykrikoides* cell-free media, strong significant negative correlations were found in *G. catenatum* between caspase-3 activity and TBARs levels (r = −0.923) at 72 h (*p* < 0.05); with the protein content a negative correlation at 24 h and 72 h (r = −0.709 and r = −0.751, respectively), was observed (*p* < 0.05). In addition, when *G. catenatum* was exposed to the cell-free filtrates of *G. impudicum*, caspase-3 activity had a positive correlation with TBARS levels at 24 h (r = 0.783) and at 48 h had a negative correlation (r = −0.709), while at 72 h there was a strong significant positive correlation (r = 0.927) (*p* < 0.05) and a negative correlation with the protein content (r = −0.737) after 72 h of exposure. 

## 3. Discussion

Allelopathy in *Gymnodinium catenatum* via cell-free media promotes oxidative stress, inducing the activation of caspase-3-like protein, involved in apoptosis processes. Evidence suggests a relationship between oxidative stress and caspase-3 activity with the growth phases. The allelopathic effect of cell-free cultures from *C. marina* var. *marina* caused the maximum O_2_^●−^ production in *G. catenatum*; the highest TBARS levels in *G. catenatum* were determined with cell-free media from *M. polykrikoides* and *G. impudicum*. Cell-free media of *G. impudicum* caused maximum SOD activity. The cell-free media of *C. marina* var. *marina* and *M. polykrikoides* caused the lowest SOD activity. The protein content in *G. catenatum* due to allelopathic effect was similar when exposed to cell-free media of *C. marina* var. *marina*, *M. polykrikoides* and *G. impudicum*. Caspase-3 activity was highest in *G. catenatum* with the cell-free media from all species. Strong positive and negative correlations were recorded between the caspase-3 activity and O_2_^●−^ production, TBARS levels, SOD activity and protein content in *G. catenatum*, due to the allelopathic effect of *C. marina* var*. marina*, *M. polykrikoides* and *G. impudicum* cell-free media.

Growth rates of dinoflagellates species vary among strain and culture conditions. The average growth rate recorded for *G*. *catenatum* of 0.57 div day^−1^ was lower compared to values reported by Band-Schmidt et al. [10] for other strains from Mexico, ~0.77 div day^−1^, and similar to those reported by Bravo and Anderson [62] with 0.56 div day^−1^ in strains from Spain (Table 3). In this study, *M. polykrikoides* registered an average growth rate of 0.59 div day^−1^, similar to the values (0.56 div day^−1^) reported by Yamatogi et al. [63] for strains from Japan, while Kim et al. [64] reported lower growth rates (0.35 div day^−1^) also in a Japanese strain. Recently, Aquino-Cruz et al. [23] reported a growth rate of 0.41 div day^−1^ for *M. polykrikoides* from a strain isolated from the coasts of Mexico. *Chattonella marina* var. *marina* had a growth rate of 0.43 div day^−1^, similar to the growth rate reported by Marshall and Hallegraeff [65], with 0.56 div day^−1^ for an Australian strain, and higher than those reported by Band-Schmidt et al. [61] of 0.30 div day^−1^ in a Mexican strain. For *G. impudicum*, a growth rate of 0.48 div day^−1^ was recorded, higher than a strain from Korea reported by Oh et al. [15] with 0.37 div day^−1^. Such differences and similarities in growth rates of strains of the same species can be due to multiple variables, such as the culture medium, photoperiod, salinity, temperature conditions or even geographical origin of the strains. 

The maximum O_2_^●−^ production was during the EEP in all the strains. Similar results were reported for *G. catenatum, C. marina* var. *marina* and *M. polykrikoides,* with higher O_2_^●−^ in the last two species [24,27,66]. In this research, *C. marina* var. *marina* and *M. polykrikoides* presented the highest O_2_^●−^ production values; however, there were no significant differences between *C. marina* var. *marina* and *G. impudicum*, and between *M. polykrikoides* and *G. catenatum*. These results suggest that other species of phytoplankton also produce high amounts of reactive oxygen species. In other studies, a high O_2_^●−^ production in *G. catenatum* was reported to be 59.7 ± 15.2 CCU per cell of O_2_^●−^ production, and total O_2_^●−^ measured in 300 µL of culture 8.0 ± 0.1 TCU × 10^4^, concluding that Gymnodiniales dinoflagellates can be potentially toxic due to O_2_^●−^ production [66]. In the phytoflagellate aggregations, ROS are generated as signaling agents, decreasing their production during the decay of cultures; they also are considered precursors of allelopathic effects [67,68]. 

Lipid peroxidation can be taken as an indicator of oxidative damage in lipids. Aquino-Cruz et al. [24] reported for *Chattonella* spp. and *M. polykrikoides* maximum TBARS values during the EEP, whereas in this study TBARS concentration increased towards the DP, especially in *M. polykrikoides*. In this study, the analyses were carried out between the EP and DP, while Aquino-Cruz et al. [24] evaluated TBARS levels only up to the EP. The results of this study can be attributed to the fact that as cells age, lipid peroxidation and mortality increase [60,69], since culture senescence is also associated with higher SOD activity. The outcome of oxidative damage is reflected in the production and integrity of proteins [69]. In this study, all analyzed species had the highest protein content in younger cultures. These results suggest a relationship in the oxidative stress as a consequence of a decreased or increased antioxidant enzyme SOD activity and glutathione. This also was reported in cyanobacteria species, *Aphanizomenon ovalisporum* and *Microcystis aeruginosa*. Cell extracts, and pure toxins microcystin and cylindrospermopsin increased the antioxidant activity in the green algae *Chlorella vulgaris* [70]; this activity can potentially act as a conservative strategy similar to that of the Antarctic cyanobacterium *Nostoc commune*, which possesses two isoforms of SOD and catalase to endure various stress conditions [71]. In higher plants, the reduced glutathione (GHS) also regulates water status and prevents chlorophyll degradation under biotic stress [72]. 

The caspase-3 activity in strains of *C. marina* var. *marina*, *M. polykrikoides*, *G. impudicum* and *G. catenatum* suggest that *M. polykrikoides* and *G. catenatum* have a shorter growth curve and a higher signal of caspase-3 activity. The maximum caspase-3 activity occurs during the DP; therefore, signaling programmed death is activated towards the end of the growth curve, probably due to a decrease of nutrients in the culture medium. Programmed cell death by nutrient decrease in phytoplankton cultures was reported in laboratory conditions in the coccolithophore *Emiliana huxyleyi* [73], and the diatom *Thalassiosira pseudonana* [74]. Nutrients may contribute, in the natural environment, to the regulating mechanism of PCD in the dinoflagellates *Karenia brevis* and *Prorocentrum donghaiense*, as a survival strategy [49,75,76].

Nutrients can affect allelopathy [77,78]. Nutrient depletion increases the toxicity of allelochemicals and their production [79]. The addition of nutrients can end allelopathy [80] and promote greater co-occurrence between phytoplankton species [36]. In this study, nutrient concentrations were not analyzed, but according to the experimental design suggested for allelopathic studies [81,82] and by Legrand et al. [34], all the experiments were carried out under optimal nutrient conditions for both the donor species of the allelopathic effect (*C. marina* var. *marina*, *M. polykrikoides*, and *G. impudicum*) and the acceptor species (*G. catenatum*). In addition, maximum exposure time (72 h) in allelopathic experiments was short, compared to the 18 or 26 days needed to research the DP in the tested species; therefore, our results can be assumed to be due to cell-free media and not nutrient-depletion media.

Allelopathy decreases algal growth, damages cell membranes and causes high mortality via lysis [33,83,84]. Mortality above 76 % associated with cells lysis caused by cell-free media, and cultures with and without cell contact was reported for *G. catenatum* [18,38]. Morphological damages were reported in vegetative cells of *Alexandrium pacificum* caused by algal allelochemicals [85]. *Cochlodinium germinatum* causes high mortality via lysis in the microalgae *Prorocentrum micans*, *Akashiwo sanguinea*, *Karlodinium veneficum*, and *Rhodomona salina* [86]. Lysis and temporary cyst formed in *Scrippsiella trochoidea* by allelopathic effects caused from cell-free media from *Karenia mikimotoi*, *Alexandrium tamarense* and *Chrysochromulina polylepis* [35,87]. Results from this study suggest that when the allelopathic effect in *G. catenatum* is more intense (i.e., higher mortality), cellular stress signals detected are lower. Greater O_2_^●−^ production in *G. catenatum* was caused by lower volumes (25 and 50 mL) of cell-free media from *C. marina* var. *marina*, *M. polykrikoides* and *G. impudicum* (Figure 4A–C).

Lu et al. [88] reported the activity of allelochemicals, finding the maximum production of ROS in the cyanobacterium *Microcystis aeruginosa* treated with the allelochemical phenol pyrogalic acid at 48 h, which was 2 times higher than what was recorded at 216 h. In this study, O_2_^●−^ production and the TBARS lipid peroxidation were consistently higher in *G. catenatum* when exposed to the lower volumes of cell-free filtrates. The SOD activity in *G. catenatum* depends on the cell-free media of the species from which it was obtained and on the exposure time (Figure 4G—I). Hong et al. [89] described the growth and the SOD activity in *M. aeruginosa* after 4 h of exposure to the allelochemical ethyl 2-methyl acetoacetate (EMA) isolated from the reed *Phragmites communis* at concentrations from 0.24 to 4 mg L ^−^^1^. This suggests that the highest O_2_^●−^ production decreased the cytoplasmic SOD activity, and the antioxidant defense may cause growth inhibition in *M. aeruginosa* from initial exposure to EMA [89]. It is probable that the decrease in SOD activity observed in *G. catenatum* in treatments with cell-free media from *C. marina* var. *marina*, *M. polykrikoides* and *G. impudicum,* which had the highest mortality, could be explained by a similar mechanism of action when maximum O_2_^●−^ production is related to the exposure time, although not necessarily to higher doses of cell-free culture media. 

The allelopathic effect associated with oxidative stress in phytoplankton species affects their photosynthetic capacity, potentially causing a decrease in the photochemical performance of photosystem II, which in turn increases the permeability of the membrane due to the oxidation of fatty acids [90,91,92]. During the interaction with the larger volumes (75 mL) of cell-free media from all donor species of the allelopathic effect, a high concentration of total proteins was recorded in *G. catenatum*. These results are consistent with those of Zhang et al. [93] who reported that higher ROS production promotes lower protein content in the dinoflagellate *Heterosigma akashiwo* exposed to 1.0 µg mL^−1^ of prodigiosin, an algaecide from bacterial origin, even when with the highest concentration treatment the protein content was similar to the controls. 

Allelochemicals and algicides increase oxidative-stress-activating pathways related to programmed cell death [73,83]. In this study, treatment of *G. catenatum* cell-free media from *C. marina* var. *marina*, *M. polykrikoides* and *G. impudicum* promoted caspase-3 activity proportional to dose-time. Similar results were reported for other phytoplankton groups exposed to allelopathic or algicidal compounds. Linoleic acid promotes the caspase-3 activity as a result of oxidative stress in *Karenia mikimotoi* [94]. Polybrominated diphenyl ethers induce oxidative stress, activating programmed cell death signals in the diatom *Thalassiosira pseudonana* [95]. During blooms of *Peridinium gutunense*, CO_2_ limitation triggers a ROS-PCD cascade reaction [83]. Also, in *M. polykrikoides*, exposure to the algicide copper sulfate and oxidizing chlorine activates a gene related with a metacaspase, a type of protease analogous to caspases [96]. 

In this study, caspase-3 activity was correlated to O_2_^●−^ production, TBARS levels, SOD activity and total protein in *G. catenatum* exposed to cell-free filtrates from *C. marina* var. *marina*, *M. polykrikoides* and *G. impudicum* (Table 2). This supports the hypothesis of oxidative stress and caspase-3 activation related to programmed cell death caused by allelopathy [8,60,83,97]. Therefore, understanding the ecological importance of programmed cell death and the relationship to chemical signaling (e.g., allelopathy) is important to understanding microscale phenomena that are reflected in the phytoplankton community [97]. 

Although there are no field studies of the allelopathic effect in *G. catenatum*, the continuous dominance in cell abundance of *C. marina* var. *marina* and *M. polykrikoides* on *G. catenatum* when these species coexist during blooms has been reported [13,14,15,17,28,98,99,100]. *Chattonella* spp. and *M. polykrikoides* were reported to promote allelopathy, inhibit growth, deform cells and cause lysis in chlorophytes, diatoms and dinoflagellates [67,101,102]. Similarly, an allelopathic effect, associated with growth inhibition, higher number of cell-chains, loss of flagella, cell deformation, swelling, prominent nucleus, rupture of cell membrane, lysis and formation of temporary cysts of *C. marina* on *G. catenatum* under laboratory conditions was reported by Fernández-Herrera et al. [38]. In addition, the allelopathic effect, including growth inhibition, cell chain fragmentation, rounded cells, loss of flagella, cell damage and lysis of *M. polykrikoides* and *G. impudicum* on *G. catenatum* was reported by Band-Schmidt et al. [18]. Results from this study could suggest a similar effect of *C. marina* var. *marina*, *M. polykrikoides* and *G. impudicum* on *G. catenatum*, potentially via allelochemicals extruded to the culture media.

The challenge for future studies is to elucidate the allelochemicals responsible for the dominance of phytoplankton species coexisting with *G. catenatum.* Bidle [8,60] proposed that programmed cell death acts as an ancestral survival strategy in the internal machinery of phytoplankton species in response to abiotic and biotic factors. Understanding the type of programmed cell death related to allelopathy can contribute to comprehend the dynamics, duration and species succession during blooms, as well as potential strategies in *G. catenatum* to survive the dominance of allelopathic species. Our results suggest that the allelopathic activity of *C. marina* var. *marina*, *M. polykrikoides* and *G. impudicum* in *G. catenatum* activates multiple oxidative stress mechanisms.

## 4. Conclusions

This study suggests that an allelopathic effect caused by cell-free media of the raphidophyte *Chattonella marina* var. *marina* and the dinoflagellates *Margalefidinium polykrikoides* and *Gymnodinium impudicum* on the toxic dinoflagellate *Gymnodinium catenatum*, potentially via allelochemicals extruded to the culture media, promotes changes in O_2_^●−^ production, lipid peroxidation levels, SOD activity and total protein content. Cell-free media from *C. marina* var. *marina* increase the caspase-3 activity in *G. catenatum* correlated with O_2_^●−^ production, TBARs levels and SOD activity. In addition, the cell-free media of *M. polykrikoides* promotes an increase of caspase-3 activity in *G. catenatum* correlated positively and negatively with O_2_^●−^ production and TBARS. The increase in the caspase-3 activity in *G. catenatum* by cell-free media of *G. impudicum* activity is correlated positively with TBARS levels. All cell-free media promoted a higher caspase-3 activity that was correlated positively with the protein content. These results support the hypothesis that the oxidative stress and the increase in the caspase-3 activity can induce programmed cell death in *G. catenatum*. 

Furthermore, results from this study suggest that the increased caspase-3 activity induces programmed cell death in *G. catenatum*. These results suggest an effect of *C. marina* var. *marina*, *M. polykrikoides* and *G. impudicum* on *G. catenatum* potentially via allelochemicals extruded to the culture media. 

## 5. Materials and Methods

### 5.1. Strains and Culture Conditions

Four monoalgal strains were used: *G. catenatum* (BAPAZ-10), *C. marina* var. *marina* (CMCV-2), *G. impudicum* (GIMP-13) and *M. polykrikoides* (MPOLY-16). Strain details are show in Table 1. All strains were cultured in modified GSe medium with the addition of earth worm extract obtained by the composte of organic waste using earthworms according to Bustillos-Guzmán et al. [103]; briefly, 50 g of earth worm humus was dissolved in 500 mL of distilled water and sterilized at 121 °C for 15 min. After 24 h it was filtered twice through GFF filters and refrigerated until its use [103]. Strains were maintained at 12/12 h light/dark cycle, ~150 µmol photons m^−1^ s^−1^ of irradiance at 24 ± 1 °C and 34 salinity. These culture conditions were maintained in all the experiments.

### 5.2. Growth Rates and Growth Curves Stages 

Growth rates and growth curves stages were determined by triplicate for each strain in 300 mL Erlenmeyer culture flasks with 150 mL of media. Growth curves were initiated with 500 cells mL^−1^ and every second day a 2.0 mL sample was fixed with lugol for cell counts. Only in the case of *Chattonella,* cells were fixed with hepes-buffered paraformaldehyde [104]. Cells were counted on 1.0 mL Sedgwick-Rafter slide under an inverted microscope (Carl Zeiss Axio Vert. A1). Cell density was used to calculate growth rates (µ) [105] according to the formula:

μ= ln (N_t_ / N_0_) / (T_i_ −T_0_) (1)

where, N_t_ and N_0_ are the total cells at the end of exponential phase (T_t_) and start of log phase (T_0_), respectively. The number of generations per day (tg) was calculated with the formula [106].

Tg = 1 / k(2)

For each species, the early exponential (EEP), late exponential (LEP), and decline phase (DP) were determined.

### 5.3. Allelopathy Experiments

Monoalgal batch cultures of *C. marina* var. *marina*, *M. polykrikoides* and *G. impudicum* were inoculated at an initial cell density of 1000 cells mL^−1^ in 1000 mL with 500 mL of medium and maintained 6 days until early exponential phase. In exponential phase, from a volume of 30 mL, cells were removed by gentle filtration using glass GF/F filters (Whatman^®^ ICT, SL, Gipuzkoa, Spain). Volumes of cell-free culture media (25, 50 and 75 mL) were recovered, these media contained cell exudates of each species, and were added to cultures of *G. catenatum* in 300 mL flasks in the following proportions (16% of cell-free media, 69% fresh GSe media and 14% of *G. catenatum* cells to 25 mL treatment), (32% of cell-free media, 52% fresh GSe media and 14% of *G. catenatum* cells to 50 mL treatment) and (48% of cell-free media, 37% fresh GSe media and 14% of *G. catenatum* cells to 75 mL treatment), all flasks with 500 cells/mL in a final volume of 150 mL, the cell free media, by triplicate. As a control, *G. catenatum* cultures were inoculated only with the GSe media and a control with their own culture medium filtrate. After 24, 48 and 72 h, from each treatment, a 2.0 mL sample was fixed with lugol for cell counts. For the determination of caspase-3-like activity, a second sample of 2.0 mL was centrifuged at 3000 rpm, the culture medium was removed, and the cell pellet was frozen at −80 °C until analyzed. The remaining volume (~146 mL) was collected in three Falcon tubes of 50 mL and placed on ice at −4 °C to be analyzed immediately. Samples from all three phases of the growth curve, on days 4, 14 and 18 for *G. catenatum* and *M. polykrikoides*, and on days 4, 24 and 26 for *C. marina* var. *marina* and *G. impudicum,* were analyzed separately.

### 5.4. Mortality

With the abundance data, the percentage of mortality (*PD*) was calculated as an indicator of the allelopathic effect, with the equation described by Fistarol et al. [35], where the number of cells of *G. catenatum* exposed to cell-free media (*D*) was related to the number of cells registered in the control (*N cont*) at the time of exposure to the cell-free filtrates (24, 48 and 72 h).

*PD* = (*D*) (100%)/*N cont* (3)

### 5.5. Superoxide Radical (O_2_^●−^) Production

Production of O_2_^●−^ was analyzed through the reduction of ferricytochrome C. The remaining volume (~146 mL) of samples was centrifuged at 2000 rpm at 24 °C; the cell pellet was recovered and re-suspended in 5.0 mL of GSe medium (cell homogenate). Then, 250 µL of the cell homogenate was transferred to a 1.75 mL conical microcentrifuge tube (Fisherbrand^TM^) and kept on ice. Cells were lysed by vortexing for 2 min. Krebs buffer (0.11 NaCl, 4.7 mM KCl, 12 nM MgSO_4_, 12 nM NaH_2_PO_4_, 25 mM NaHCO_4_, and 1 mM glucose) and cytochrome-C (15 µM) were added. Tubes were capped and incubated at 37 ± 1 °C during 15 min in a shaking water bath (Polyscience, Niles, IL, USA). N-ethylmaleimide (3 nM) was added, and the homogenate was shaken to stop the reaction. Tubes were centrifuged at 3000 rpm, 4 °C for 10 min. Supernatant was transferred to polystyrene disposable cuvettes (Fisherbrand^TM^), and absorbance was recorded at 560 nm in a spectrophotometer (Beckman Coulter DU 800, Fullerton, CA, USA). A blank without homogenate was included for each sample. Superoxide radical production was calculated, according to the following formula [107]:

O_2_^●−^ = Abs (Sample − Blank)/21 nmol/L cm = nmoles O_2_^●−^/min mL (4)

### 5.6. Thiobarbituric Acid Reactive Substances (TBARS) Levels

Hydroperoxides and aldehydes resulting from lipid peroxidation in the sample were analyzed by the reaction of 2-thiobarbirutic acid (TBA) to form malondialdehyde (MDA), following the method of Persky et al. [108] and Zenteno-Savín et al. [109]. In a 1.7 mL conical microcentrifuge tube (Fisherbrand^TM^), 500 µL of the cell homogenate was incubated at 37 °C in a shaking water bath (Polyscience). Tubes were placed on ice and in a solution of trichloroacetic acid (TCA 20 %), and HCl (1.0 M) was added to stop the reaction, followed by the addition of TBA 1%, and vortexed. Samples were incubated at 90 °C in a shaking water bath for 10 min, followed by 1 min on ice and centrifuged at 3000 rpm (1500× *g*) for 10 min at 4 °C. The absorbance of the supernatant was recorded in a spectrophotometer (Beckman Coulter DU 800, Fullerton, CA, USA) at 530 nm. Calculations for TBARS concentration in cells were done adjusting the values to a standard curve of 1,1,3,3-tetraethoxypropane (TEP), in concentrations that ranged from 0 to 5 mmol 250 µL^−1^, and the result was expressed in nmol 10^−4^ cells^−1^.

### 5.7. Superoxide Dismutase (SOD) Activity

Superoxide dismutase activity was determined using the xanthine/xanthine oxidase system as O_2_^●−^ generator, when it reacts with nitroblue tetrazolium (NBT), reducing it and producing formazan. This chemical species can be detected by spectrophotometry, when SOD inhibits the reduction of NBT [110].

An aliquot of 250 µL of the cell homogenate was mixed with 500 µL of homogenizing solution (phosphate buffer 0.1M, EDTA 60 mM and phenyl methyl sulfonyl fluoride PMSF); samples were centrifuged at 300 rpm, during 15 min at 4 °C. Supernatant was recovered and the precipitate was discarded. Working solution containing sodium-carbonate buffer (50 mM), xanthine (0.1 mM), NBT (0.025 mM), EDTA (0.1 mM,) xanthine oxidase (XO, 0.1 U mL^−1^) and blank or sample were mixed. The absorbance was recorded at 560 nm every 30 s for 5 min (∆A560). SOD activity was expressed in Units mg^−1^ of protein 10^−4^ cells.

### 5.8. Total Protein

The amount of total proteins was determined by the Bradford method [111]. This method is based on the reaction of Coomassie brilliant blue (Bio-Rad^®^ inc, Hercules, CA, USA.) with the basic amino acid residues, especially arginine. Phosphate buffer (0.1M) and EDTA (60 mM) were added to an aliquot of 250 µL the cell homogenate; samples were mixed in a vortex for 2 min, centrifuged at 3000 rpm for 15 min at 4 °C and the supernatant was recovered. In a 96-well microplate, samples and the standard curve with bovine serum albumin (BSA) at concentrations from 0.005 to 0.2 mg mL^−1^ of distilled water were mixed with the colorant and the microplate was shaken gently for 30 s. The microplate was covered and incubated at 25 °C. The absorbance was determined at 620 nm in a microplate reader (Thermo-Scientific).

Results were expressed in mg mL^−1^ proteins 10^−4^ cells.

### 5.9. Caspase-3 Activity

Apoptotic activity was determined using the Enzchek Caspase-3 Assay Kit #2 (Invitrogen). In triplicate, cell homogenates were centrifuged at 3000 rpm in 2.0 mL microcentrifuge tubes (Eppendorf®), the culture medium was removed, and the cell pellet was frozen at −80 °C. Each pellet was resuspended in the lysis buffer, stirred during 2 min in a vortex, frozen and thawed twice; cell pellet lysates were centrifuged at 5000 rpm, during 5 min at 4 °C. The supernatant was recovered and transferred to 96-well microplates, Z-DEVD-R110 (5 mM) substrate in solution with 2x reaction buffer added to all wells. Samples were incubated for 2 h in darkness. The fluorescent signal of rhodamine (R-110) whit substrate-enzyme Z-DEVD-R110 was subsequently determined using a microplate reader (Ex 485 nm; Em: 520 nm). Lysis buffer (1X) and GSe medium were used as a negative control. The moles produced in the reactions with the activity of caspase-3 were calculated in relative fluorescent units (RUF) h^−1^ mg^−1^ protein. Prior to adding the substrate, cell pellets were incubated for 30 min with reversible caspase inhibitor Ac-DEVD-CHO (5 mM) to confirm that the observed fluorescence corresponded to the activity of the caspase-3 proteases. [112,113].

### 5.10. Statistics Analysis

Kolmogorov–Smirnov, Shapiro–Wilk normality tests and Levene test homoscedasticity were performed on all data. For growth phases and allelopathic experiments, a one-way analysis of variance (ANOVA) (*p* < 0.05) with a post hoc Tukey HSD (Honest Significant Difference) were applied. A Pearson correlation analysis was performed to evaluate the relationship between PCD and ROS. All statistical analyses were done using Statistica StatSoft^®^ (Tulsa, OK, USA) software.

## Figures and Tables

**Table 1:** Strains utilized in allelopathy experiments, collection site, growth rate and generations per day.

Class/Species	Strain	Collection Site/Year	Growth Rate div/day	Generations per Day
Dinophyceae				
*Gymnodinium catenatum*	BAPAZ-10	Bahía de La Paz, B.C.S. 2017	0.57	0.52
*Margalefidinium polykrikoides*	MPOLY-16	Bahía Concepción, B.C.S. 2017	0.59	0.50
*Gymnodinium impudicum*	GIMP-13	Bahía Concepción, B.C.S. 2013	0.48	0.69
Raphidophyceae				
*Chattonella marina* var. *marina*	CMCV-2 Band-Schmidt et al. [61]	Bahía Concepción, B.C.S. 2000	0.43	0.61

**Table 2:** Correlation between caspase-3-like activity and the oxidative stress indicators in *Gymnodinium catenatum* exposed to allelopathic cell-free media of *Chattonella marina* var. *marina*, *Margalefidinium polykrikoides* and *Gymnodinium impudicum*.

Caspase-3 Activity	h	O_2_^●−^	TBARs	SOD	Protein
Control	24	0.112	0.475	−0.654	−0.891 *
Control	48	0.019	0.031	0.599	−0.872 *
Control	72	−0.720	−0.026	0.327	−0.832 *
*C. marina* var. *marina*	24	0.192	0.682	−0.852*	−0.731 *
*C. marina* var. *marina*	48	−0.796 *	−0.495	−0.733 *	−0.739 *
*C. marina* var. *marina*	72	−0.707 *	−0.927 *	−0.055	−0.215
*M. polykrikoides*	24	−0.626	0.567	−0.006	−0.709 *
*M. polykrikoides*	48	−0.091	−0.360	0.259	−0.751 *
*M. polykrikoides*	72	−0.548	−0.923 *	−0.048	−0.319
*G. impudicum*	24	−0.106	0.783 *	0.259	−0.595
*G. impudicum*	48	0.036	−0.709 *	0.161	−0.737*
*G. impudicum*	72	0.072	0.927 *	0.489	0.011

O_2_^●−^, Superoxide radical production; TBARS, thiobarbituric acid reactive substances; SOD, superoxide dismutase activity, total protein concentration. * Marked correlation are significance at *p* < 0.05 (*n* = 12).

**Table 3:** Growth rates, culture condition and location of isolation of some strains of *Gymnodinium catenatum*, *Margalefidinium polykrikoides*, *Chattonella marina* var. *marina* and *Gymnodinium impudicum*.

Species/Strain	Growth Rate (div day^−1^)	Medium Culture	Temperature (°C)	Salinity	Irradiance (µmol photons m^−1^ s^−1^)	Cycle (Light/Dark)	Location	Reference
*Gymnodinium catenatum*/ BAPAZ-10	0.57	GSe with soil extract	24	34	~150	12:12	Gulf of California	This study
*Gymnodinium catenatum*	0.56	K	22–28	-	150	14:10	Spain	Anderson and Bravo [62]
*Gymnodinium catenatum*	~0.77	GSe and f/2	20–29	30–35	150–230	12:12	Gulf of California/Mexican Pacific	Band-Schmidt et al. [10]
*Margalefidinium polykrikoides*/MPOLY-16	0.59	GSe with soil extract	24	34	~150	12:12	Mexican Pacific	This study
*Margalefidinium polykrikoides*	0.41	f/2	15–30	20–36	30–238	12:12	Japan	Kim et al. [64]
*Margalefidinium polykrikoides*	0.56	ESM	27.5	32	80	14:10	Japan	Yamatogi et al. [63]
*Margalefidinium polykrikoides*	0.41	GSe with soil extract	24	34	~150	12:12	Gulf of California	Aquino-Cruz et al. [23]
*Chattonella marina* var. *marina*/CMCV-2	0.43	GSe with soil extract	24	34	~150	12:12	Mexican Pacific	This study
*Chattonella marina*/CMPL01	0.47	GSe	10–35	10–50	150	12:12	Australia	Marshall and Hallegraeff [65]
*Chattonella marina/*CSCV-1	0.30	f/2	30	-	150	12:12	Mexican Pacific	Band-Schmidt et al. [61]
*Gymnodinium impudicum/*GIMP-13	0.48	GSe with soil extract	24	34	150	12:12	Mexican Pacific	This study
*Gymnodinium impudicum*	0.37	L1 limited phosphorus	20	30	300	12:12	Korea	Oh et al. [15]

## Data Availability

Not applicable.
